# An Integrated Metabolomic Screening Platform Discovers the Potential Biomarkers of Ischemic Stroke and Reveals the Protective Effect and Mechanism of Folic Acid

**DOI:** 10.3389/fmolb.2022.783793

**Published:** 2022-05-18

**Authors:** Yan-hui Yang, Lei Lei, Yin-ping Bao, Lu Zhang

**Affiliations:** ^1^ Department of Clinical Nutrition, The Second Affiliated Hospital, Harbin Medical University, Harbin, China; ^2^ Department of Nutrition, Harbin First Hospital, Harbin, China; ^3^ Department of Obstetrics and Gynecology, The Second Affiliated Hospital, Harbin Medical University, Harbin, China; ^4^ Department of Clinical Nutrition, Heilongjiang Provincial Hospital, Harbin, China

**Keywords:** metabolomics, metabolites, mass spectrometry, biological pathway, potential biomarkers

## Abstract

Folic acid has a protective effect against ischemic stroke. However, the protective pharmacological mechanism remains unclear. The aim of this study is to explore the protective effect of folic acid on ischemic stroke animals by an integrated metabolomic biomarker screening platform. Based on ultra-performance liquid chromatography-tandem mass spectrometry (UPLC/MS) coupled with multivariate data analysis, the changes in metabolites and pathways were characterized. We found that the metabolic alteration involved a total of 37 metabolites, of which 26 biomarkers such as γ-aminobutyric acid, lysine, glutamate, ribose, and valine can be regulated by folic acid *via* metabolic pathways of amino acid metabolism, carbohydrate metabolism, fatty acid metabolism, citrate cycle, and pyruvate metabolism, which may be the potential therapeutic targets of folic acid against ischemic stroke. Folic acid as an emerging potential natural anti-fibrosis agent has significant activity in protecting against middle cerebral artery occlusion-induced rat ischemic stroke model by delaying pathological development, reversing the metabolic biomarkers, and mainly regulating the perturbation in amino acid metabolism, carbohydrate metabolism, fatty acid metabolism, citrate cycle, and pyruvate metabolism. It also showed that the integrated metabolic biomarker screening platform could provide a better understanding of the therapeutic effect and mechanism of drugs.

## Introduction

With more than 800,000 cases occurring every year, stroke has become the fifth leading cause of death in the United States (Feigin et al., 2016; Jamthikar et al., 2021). Among them, ischemic stroke (IS) is the most common type of stroke accounting for 87%, which caused irreversible damage through a variety of cerebral blood supply disorders to the local brain tissue (Sümer and Özön, 2017; Du et al., 2020; Qin et al., 2020; Tan et al., 2020; Salazar-Camelo et al., 2021). In clinical practice, conventional treatment measures, such as oxygen inhalation, controlling infection, and lowering intracranial pressure, and specialized treatments, such as early thrombolysis, antiplatelet, anticoagulation, and defibrosis, have certain neurological side effects (Amr et al., 2016; Sarmiento et al., 2019; Cheng et al., 2020; Herpich and Rincon, 2020; Hill, 2020). In addition, clinically available drugs cannot meet the increasing clinical demand (Zhang A. et al., 2018; Chen et al., 2020).

Some studies showed that herbal medicine has a vital role for health promotion and disease prevention (Wang et al., 2014; Zhang et al., 2017; Zhang et al., 2019a). Folic acid (FA) is a water-soluble natural compound found in food sources. It plays an essential role in deoxyribonucleic acid and ribonucleic acid biosynthesis (Hsu et al., 2018). FA helps to promote the growth and reproduction of body cells, and can also be applied as a substitute for calcium leucovorin as adjuvant therapy for methanol toxicity. Some studies have shown that FA significantly reduces risk of IS (Sato et al., 2002; Xia et al., 2014; Wise, 2015; Jadavji et al., 2018; Dong et al., 2020; Preeti et al., 2020).

As an emerging platform of system biology, metabolomics has the ability to obtain endogenous metabolites to assess their pharmacology, toxicology, drug safety, and efficacy (Zhang et al., 2014a; Nan et al., 2016; Zhang et al., 2016; Li et al., 2017). It is a powerful tool to understand the therapeutic mechanism of natural products at the metabolic level (Li et al., 2016; Xie et al., 2019; Zhang et al., 2019b). Furthermore, high-throughput technology, including nuclear magnetic resonance spectroscopy, gas chromatography mass spectrometry, and liquid chromatography mass spectrometry (LC-MS), combined with multivariate analysis was also performed in metabolomics research (Zhang et al., 2012; Zhang et al., 2014b; Liang et al., 2016a; Liang et al., 2017; Wang et al., 2019; Guo et al., 2020; Zhang et al., 2020). In this study, we detected the metabolite and metabolic pathway changes in brain tissue of a middle cerebral artery occlusion (MCAO) IS rat model and explored the protective effect and mechanism of FA by using an integrated metabolomics platform.

## Materials and Methods

### Chemicals and Reagents

Chromatographic grade acetonitrile, methanol, and formic acid were obtained from Fisher Scientific (Fair Lawn, NJ, United States). Ultrapure water was obtained from a Milli-Q purification system (Billerica, MA, United States). FA (PurityAbove 90%, Lot No. 1007456) and nimodipine tablets (Batch No. BJ29160) were purchased from Changbaishan Pharmaceutical Co. Ltd. (Jilin, China) and Bayer Healthcare Company Ltd. (Leverkusen, Germany), respectively. Physiologic saline solution and anesthetic were supplied from Chengdu Mansite Bio-technology Co., Ltd. (Sichuan, China). ELISA kits of nitric oxide (NO), superoxide dismutase (SOD), malondialdehyde (MDA), and nitric oxide synthase (NOS) were purchased from Chengdu Mansite Bio-technology Co., Ltd. (Sichuan, China). ELISA kits of tumor necrosis factor-α (TNF-α), interleukin-6 (IL-6), and interleukin-1 beta (IL-1β) were bought from Sigma-Aldrich (St Louis, MO, United States). ELISA kits of vascular endothelial growth factor (VEGF), hypoxia inducible factor-1α (HIF-1α), and matrix metallopeptidase 2 (MMP-2) were provided by Carlo Erba (Val de Reuil, France). Standard substance leucine enkephalin (Purity99.4%, Lot No. 1912032) was obtained from Lonza (Barcelona, Estado Anzoátegui, Spain).

### Animals

All animal care and experimental procedures described herein were approved by the Institutional Animal Care and Use Committee of Harbin Medical University. A total of forty-eight healthy male Wistar rats (10–12 weeks old, weighing 180–220 g) were procured from the Experimental Animal Center of Beijing University of Chinese Medicine (Beijing, China). Animals were housed in a controlled breeding room under the following conditions: a constant temperature of 24 ± 3°C, 60 ± 5% relative humidity, and a 12-h light/dark cycle. All the rats had *ad libitum* access to food and water.

### Animal Model

After acclimatization for 1 week, rats were stochastically allocated into six groups with eight animals in each group, including the sham operation group (SHA group), IS model group (MOD group), nimodipine group (NIM group), low dosage of FA group (L-FA group), middle dosage of FA group (M-FA group), and high dosage of FA group (H-FA group). The animal model is carried out by middle cerebral artery occlusion method (MCAO). Briefly, all rats were firstly anesthetized with 10% chloral hydrate (350 mg/kg) after fasting for 12 h, and then the common carotid artery (CCA), internal carotid artery (ICA), and external carotid artery (ECA) were exposed using blunt dissection. A 4–0 surgical nylon suture with a heat-rounded tip was inserted into the lumen of the ICA from the ECA and advanced 20–25 mm as the distance from the bifurcation to prevent the source of the right middle cerebral artery. After 1.5 h, further reperfusion was produced by withdrawing the monofilament. Rats in the SHA group were subjected to the same procedures except for the insertion of nylon monofilament. During the operation, environment temperature was controlled at 37°C and vital signs such as heart rate and blood pressure were checked. Upon waking up, the left forelimb of the animal is weak and the animal crawls to the left, or crawls to the left in a circle without irritability; at this point, one can preliminarily judge whether the model is successful.

### Drug Treatment

Five days before the induction of IS, animals in each group begin to receive drug treatment. The period of administration is 20 days. The SHA and MOD groups were administered 0.9% normal saline daily by gavage; meanwhile, rats in the NIM, L-FA, M-FA, and H-FA group were treated with nimodipine tablet and FA at a dose of 24, 70, 140, and 280 mg/kg every day, respectively.

### Neurological Function Evaluation

At 24 h after reperfusion as previously described, neurological functional deficiency scores of rats were assessed in line with Longa’s five-point scale: zero points: no neurobehavioral dysfunction; one point: failure to extend the contralateral front limb completely; two points: circles counterclockwise; three points: turns around to the other side seriously; four points: cannot walk spontaneously. The evaluation criterion is that the higher the score, the more serious the impairment of animal behavior. The same test operation is performed on the 7th and 14th day after cerebral ischemia reperfusion.

### Beam Walking Test

The beam walking test for monitoring motor function of animals was carried out 15 days after modeling. Rats in each group were trained to walk across a narrow wooden beam that is set 4 cm wide, 105 cm long, and 80 cm above the ground. The starting area was deemed as the front 20 cm on the beam, and a horizontal line was delimited at a distance of 20 cm from the starting zone. When the rat starts moving from the starting area, total times consumed to travel across the beam were recorded; the animal needs to be pre-trained three times a day for 2 continuous days before the test.

### Clinical Biochemistry Detection

Twenty-four hours after the final time of drug treatment, rats from the SHA, MOD, NIM, L-FA, M-FA, and H-FA groups were mildly anesthetized with 50 mg/kg sodium pentobarbital intraperitoneally. Blood samples were collected from the abdominal aorta, which were placed 5 min for coagulation and then were centrifuged at 3,500 rpm at 4°C for 10 min. The supernatants were transferred into a new plastic tube and stored at −80°C. Before clinical biochemistry detection, serum samples were thawed at 4°C to indoor temperature. In light of the manufacturer’s instructions, serum samples were dissolved by the corresponding solution and measured by an automatic biochemical analyzer.

### Brain Tissue Preparation

After blood collection, each group of rats was quickly decapitated, and then brain tissue was removed, weighed, frozen in liquid nitrogen, and stored at −80°C until use. The frozen brain was weighted and delivered into an Eppendorf tube. Ice-cold high-purity methanol/water mixture (v/v is 80/20) was added for metabolite extraction to achieve 30 μl/mg of tissue. The suspension was homogenized with an ultrasound sonicator (Qsonica, CT, United States) for 2 min, then stored at −20°C for 1 h, and centrifuged at 13,000 rpm, at 4°C for 10 min. The obtained supernatants were collected and transferred into a new Eppendorf tube; all the samples were evaporated to dryness for 6 h. For LC-MS analysis, the dried samples were reconstituted with 0.1 ml of 70% MeOH with 0.01% formic acid. After centrifugation at 13,000 rpm, at 4°C for 5 min, the prepared samples were filtered through a 0.22-μm micropore filter. In addition, 48 brain tissue samples randomly selected from each sample (10 μl) from these six groups were mixed together as quality control samples to validate the stability of the UPLC-MS system, and were subjected to the same procedures as above.

### Metabolomics Analysis

Online UPLC-MS analysis was performed using a Vanquish Duo UPLC system (Thermo Fisher Scientific, San Jose, CA, United States) and a 6,520 quadrupole time-of-flight mass spectrometer equipped with an electrospray ionization (ESI) source (Agilent, San Jose, CA, United States). Sample separation was achieved using an ACQUITY UPLC HSS T3 column (100 mm × 2.1, 1.8 μm; Waters Corporation, Milford, United States). The UPLC parameters were set as follows: column temperature, 35°C; flow rate, 0.3 ml/min; injection volume, 4 μl; autosampler temperature, 4°C. The mobile phase consisted of acetonitrile containing 0.05% formic acid (phase A) and water containing 0.05% formic acid (phase B); the elution gradient is as follows: 0–2 min, 2%–10% A; 2–4 min, 10–25% A; 4–8 min, 25%–50% A; 8–9 min, 50%–99% A; 9–11 min, maintain 99% A; 11–13 min, linear decrease from 99% to 2% A; keep at 2% A for 2 min for column equilibration. Methanol as blank injection was run after every four samples. The mass range is 50–1,500 m/z in the centroid mode with 35,000 mass resolution for full-scan analysis.

The ESI source parameters were as follows: electrospray capillary voltage is 4.0 kV in ESI+ mode and 3.0 kV in ESI- mode, ion spray voltage is 3.2 kV, ion source temperature is 400°C, curtain gas is 38 psi, declustering potential (DP) is 70 V, collision energy (CE) is 45 eV, collision energy spread (CES) is 25 eV, gas temperature is 280°C, gas flow rate is 13 L/min, and nozzle voltage is 2,300 V in both ion modes. A reference mass solution of 0.20 ng/ml leucine enkephalin was continuously infused using an isocratic pump connected to a dual sprayer transferring into the electrospray ionization source (ESI + ion mode, 556.2771 m/z; ESI- ion mode, 554.2614 m/z). Before brain tissue sample analysis, QC samples were investigated six times for the UPLC/MS system balance, and then every eight samples were examined to determine whether the systematic error is within the controllable range.

All the raw UPLC-MS data of brain tissue were acquired by MassHunter Acquisition software (version 5.0, Agilent Technologies, Santa Clara, CA). The matched peaks in the original spectral data usually differ in their charge-to-mass ratio and retention time. High-resolution mass spectrometry usually requires the mass measurement accuracy of the instrument to be less than 10 ppm. Center scaling is the process of subtracting the average value of each variable from the metabolome data. The intensity of each ion from samples was normalized though a comparison of the total ion intensity of peaks in MS profiles, in which the resultant dataset (csv file) was imported into SIMCA (version 13.0, Umetrics, Sweden) for multivariate statistical analysis such as unsupervised principal component analysis (PCA) and supervised orthogonal partial least squares discriminant analysis (OPLS-DA).

From variable importance in the projection (VIP) scatter plot calculated from the OPLS-DA model. Candidate biomarkers were selected by ions meeting the following criteria: VIP of more than 1.5 and *p* values of <0.05 in Student’s *t*-tests. By searching accurate m/z values of the peaks from the available biochemical databases, such as HMDB, KEGG, and Chemspider, an endogenous metabolite structure was initially identified. Combined with the comparison of MS/MS spectra, retention times, and reference substance, the differential metabolites were further confirmed, in which the exact m/z values and fragment ion intensities from the acquired MS/MS spectra of potential metabolites must be in accordance with those of reference metabolites or the database.

### Statistical Analysis

The statistical analysis was performed using two-tailed Student’s *t*-tests for group comparison and repeated measures analysis of variance (ANOVA) followed by the Bonferroni procedure, which was conducted with software SPSS 23.0 (International Business Machines Corporation, Armonk, NY, United States). All the data are expressed in mean values ± standard deviation. Differences with *p* < 0.05 were considered statistically significant.

## Results

### Neurological Deficit, Motor Function, and Clinical Biochemistry Analysis

**FIGURE 1:**
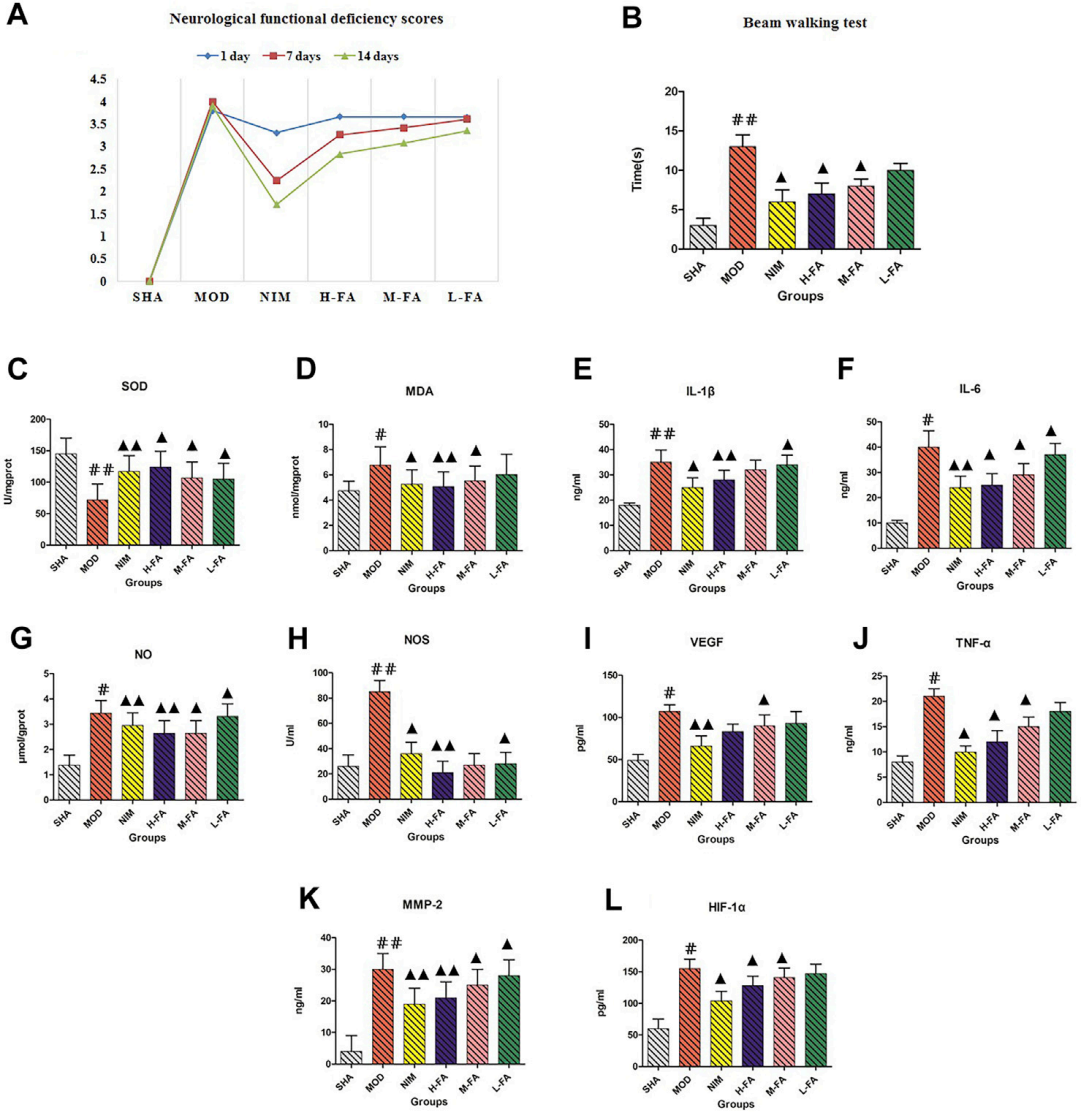
The comparison of neurological functional deficiency scores, total times in beam walking test, and chemical indicator changes between the SHA, MOD, NIM, L-FA, M-FA, and H-FA groups (*n* = 8 in every group). Data with statistical significance are represented as "#" or “▲” with a *p*-value less than 0.05. Data with statistical significance are represented as "##" or “▲▲” with a *p*-value less than 0.01.

Results of neurological functional deficiency score test showed that the neurological deficit scores of rats in the MOD group were greatly higher than those in the SHA group. Treatment with FA did not relieve the deficiency at 1 day after reperfusion, and then the scores were significantly lowered by FA treatment at 7 and 14 days after reperfusion (Figure 1A). From the results of the beam walking test, rats in the MOD group spent a longer time traveling across the beam compared with the SHA group. Rats treated with FA 20 days spent a shorter time walking across the whole beam compared with the MOD group, suggesting that exercise and coordination capacity are gradually restored as the dosage of FA increased (Figure 1B). From Figure 1C to 1L, it is easy to see that the MDA, NO, TNF-α, IL-1β, IL-6, VEGF, HIF-1α, NOS, and MMP-2 concentrations in brain tissue were increased, and the concentration of SOD was decreased compared with rats in the SHA group. After the therapeutic period of FA, SOD level in rats was significantly higher than those in the MOD group (*p* < 0.01). Meanwhile, the MDA, NO, TNF-α, IL-1β, IL-6, VEGF, HIF-1α, NOS, and MMP-2 levels were downregulated with significant statistical implications, in which brain MDA, NO, IL-1β, NOS, and MMP-2 levels obviously decreased. Compared with the MOD group, the change in the trend of physical and biochemical indicators in the NIM group and the different dosages of the FA group is similar, indicating that FA has a certain therapeutic effect on IS mediated by improving neurological deficit, reducing oxidative damage and inflammation, and promoting motor function and angiogenesis. The efficacy of FA on IS was performed in a dose-dependent manner.

### UPLC-MS Method Validation

It is necessary for a large-scale metabolomics study to ensure good repeatability and stability in an analytical platform, excluding the major differences noticed among groups, which give rise to instability in the analytical method. After raw data preprocessing, ions that were investigated in <50% of the QC samples were eliminated from the dataset. Two datasets were extracted from 48 brain tissue samples containing 1,483 ions in positive mode and 1,256 ions in negative mode. The repeatability and stability of the analytical platform of the remaining ions were determined by relative standard deviation (RSD%) calculation; 84.12% and 86.76% of the peaks monitored in both ion mode had RSD values less than 15%, indicating that the developed UPLC-MS method has repeatability and stability.

### Metabolite Profiling in Brain Tissue

**FIGURE 2:**
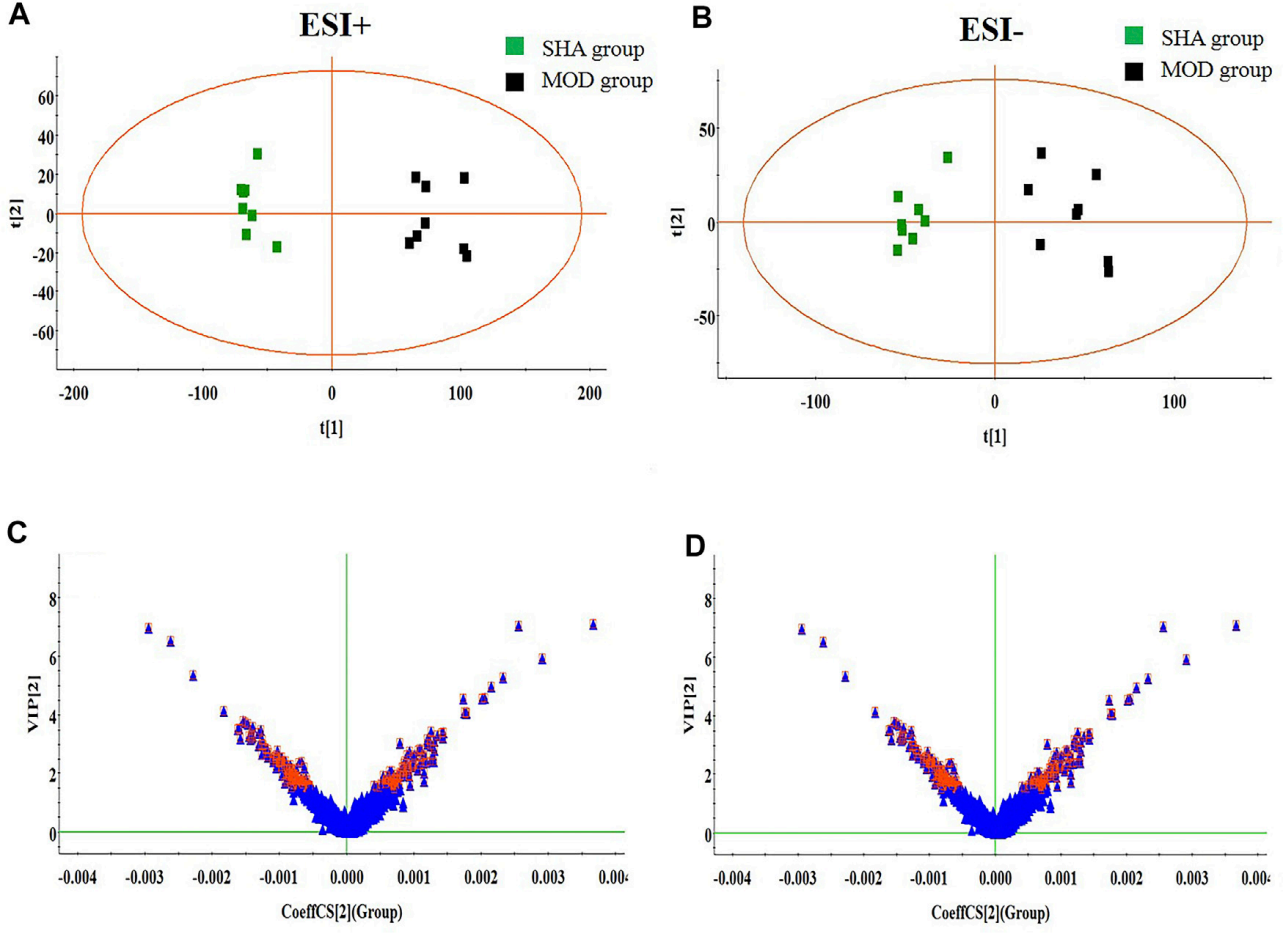
OPLS-DA score plot and VIP-plot between the SHA and MOD groups (*n* = 8 in every group). **(A)** and **(C)** are in positive ion mode; **(B)** and **(D)** are in negative ion mode.

**FIGURE 3:**
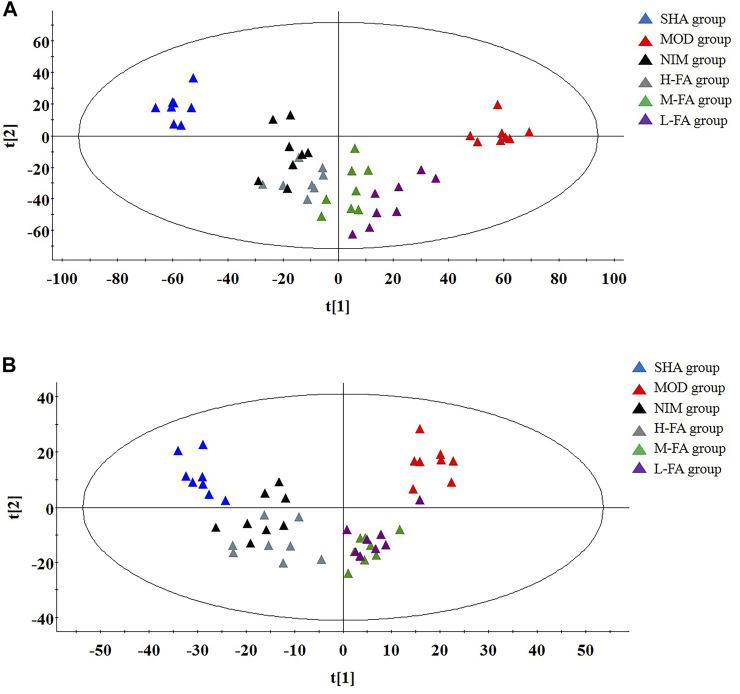
In positive ion mode: PCA score plots present a separate trajectory of metabolites in brain tissue among the SHA, MOD, NIM, L-FA, M-FA, and H-FA groups (*n* = 8 in every group) **(A)**. In negative ion mode: PCA score plots present a separate trajectory of metabolites in brain tissue among the SHA, MOD, NIM, L-FA, M-FA, and H-FA groups **(B)**.

The metabolites of brain tissue were assessed by the validated UPLC-MS method, in which the representative total ion chromatogram of six groups in both positive and negative ion modes is well separated in 9 min. Though a slight discrepancy was noticed among the above-mentioned groups, visual examination was not sufficient to explain the metabolic differences in the SHA, MOD, NIM, L-FA, M-FA, and H-FA groups. Multivariate statistical analysis was further applied to probe into the therapeutic effect of FA. Firstly, the brain metabolic difference between the SHA group and MOD group was highlighted by the score plot of the OPLS-DA model with Pareto scaling; the R^2^Y and Q^2^ values of 0.982 and 0.937 in positive ion mode and 0.966 and 0.925 in negative ion mode imply the outstanding fitness and prediction capability of the constructed OPLS-DA model. No over-fitting was observed in the light of the results of the permutation test; the R^2^Y-intercepts and Q^2^-intercepts were 0.805 and −0.210, respectively, which were lower than the original values, indicating the great predictability and goodness of fit of the established model. Clear separation was easily observable from the OPLS-DA score plot in the first principal component between the SHA group and MOD group, indicating that the brain tissue metabolic pattern has been altered after MACO surgery, in which each spot represented a sample (Figures 2A,B). VIP plot diagrams of OPLS-DA were generated to better understand the ion contribution for group difference. The greater contribution to the clustering of the SHA group and MOD group, the further away they were from the origin (Figures 2C,D). In PCA score plots of SHA, MOD, NIM, L-FA, M-FA, and H-FA groups, they present clear parting. The cluster of NIM, L-FA, M-FA, and H-FA groups shows the tendency to approach the SHA group, far from the MOD group, which indicates that FA has a protective effect on MACO rats in line with the results of neurological deficit, motor function, and clinical biochemistry. Meanwhile, with the concentration of FA increased, the distance between the SHA group and the FA group gradually decreases (Figures 3A,B).

### Biomarker Analysis

**FIGURE 4:**
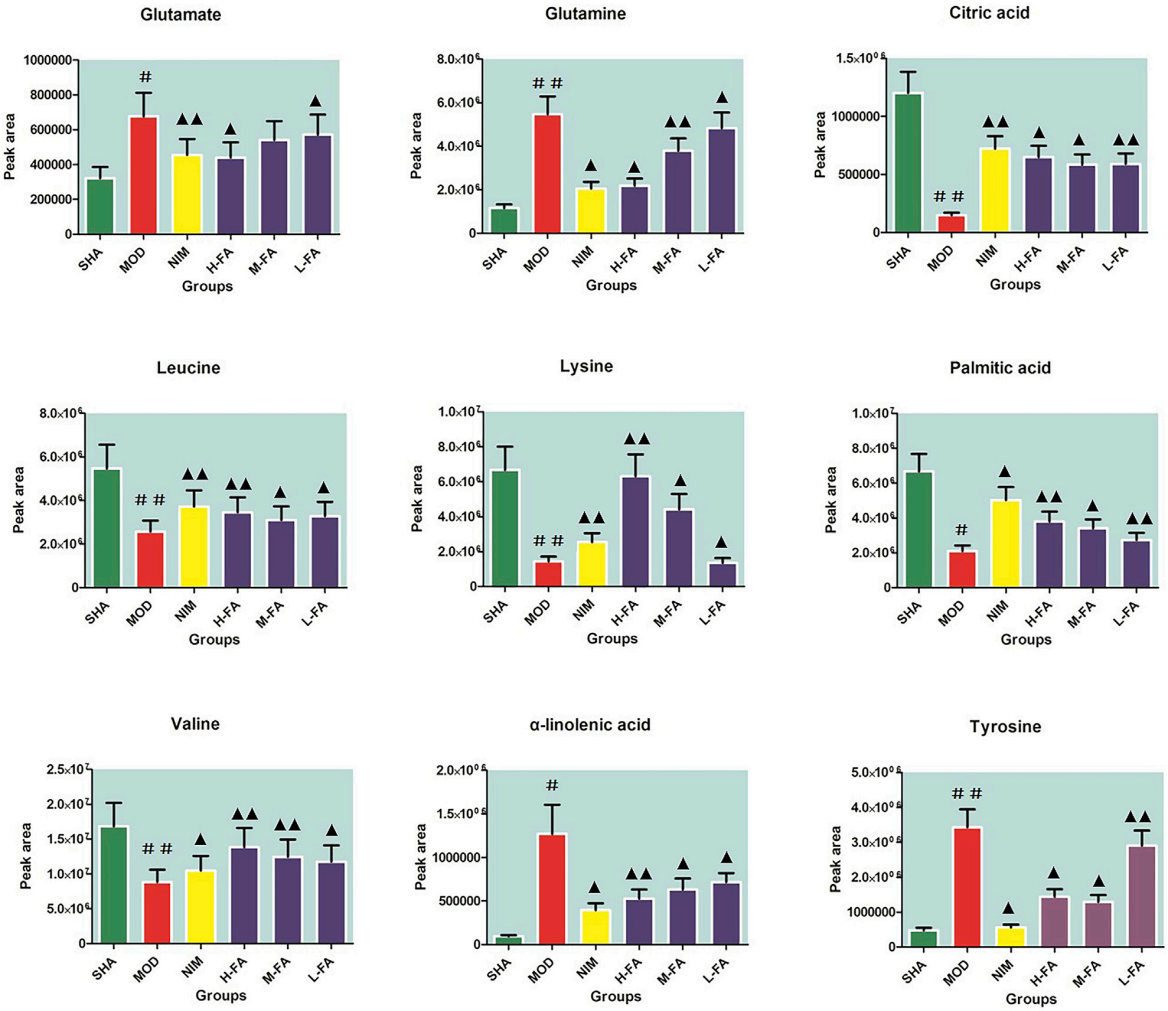
The relative signal intensity calculation of common peaks in the SHA, MOD, NIM, L-FA, M-FA and H-FA groups as a semi-quantitative comparison objector (*n* = 8 in every group). Data with statistical significance are represented as “#” or “▲” with a *p*-value less than 0.05. Data with statistical significance are represented as “##” or “▲▲” with a *p*-value less than 0.01.

Candidate biomarkers were selected by metabolite ions in brain tissue that contribute to the distinction between the SHA and MOD group that meet the following selection criteria: VIP values were larger than 1.5 gained from the OPLS analysis plot and then further filtered by *p*-values less than 0.05 calculated using Student’s *t*-test. A total of 37 endogenous metabolites, including seventeen kinds of amino acids, seven kinds of organic acids, five kinds of fatty acid, three kinds of carbohydrates, three kinds of lipids, and other metabolites such as sphingosine and pyruvic acid, were identified with the help of accurate MS and tandem MS information of the peaks from available biochemical databases and reference substances, emphasizing pathological changes in rats after MACO surgery. Details on ion mode, Rt (min), chemical name, formula, and HMDB code are listed in Table S1. Compared with the SHA group, animals in the MOD group had elevated levels of γ-aminobutyric acid, glycine, glutamate, methionine, tyrosine, serine, lactic acid, phenylalanine, glutamine, α-hydroxybutyrate, arachidonic acid, homocysteine, arginine, alanine, α-linolenic acid, docosahexaenoic acid, and acetylcarnitine and lowered levels of lysine, taurine, ribose, pyruvic acid, valine, creatine, leucine, glucose, isoleucine, phosphocreatine, citric acid, glucose 6-phosphate, hippuric acid, choline, LysoPC(20:3), LysoPC(17:0), palmitoleic acid, palmitic acid, sphingosine, and LysoPC(20:1), implying a marked metabolic perturbation in rats with IS. After FA treatment, nine metabolites in the L-FA group were partly recovered and close to the level of the SHA group. With the increasing concentration of FA in the M-FA group, fifteen metabolite levels, such as γ-aminobutyric acid, glucose, hippuric acid, and phenylalanine, improved. When IS rats were administered with a high dosage of FA, metabolic profiling change close to the SHA group reached optimum values, and twenty-six metabolites, namely, γ-aminobutyric acid, lysine, glutamate, ribose, pyruvic acid, valine, leucine, tyrosine, glucose, isoleucine, citric acid, glucose 6-phosphate, lactic acid, hippuric acid, phenylalanine, glutamine, α-hydroxybutyrate, arachidonic acid, homocysteine, arginine, LysoPC(20:3), alanine, LysoPC(17:0), palmitic acid, LysoPC(20:1), and α-linolenic acid, were effectively called back. A heat map was established in Supplementary Figure S1 according to the relative quantities of each metabolite to illustrate the tendency of biomarker variation among six groups. The relative signal intensities of common in different groups as semi-quantitative comparison objector were calculated and shown in Figure 4 for giving prominence to FA pharmacological activity.

### Metabolic Pathway Analysis

**FIGURE 5:**
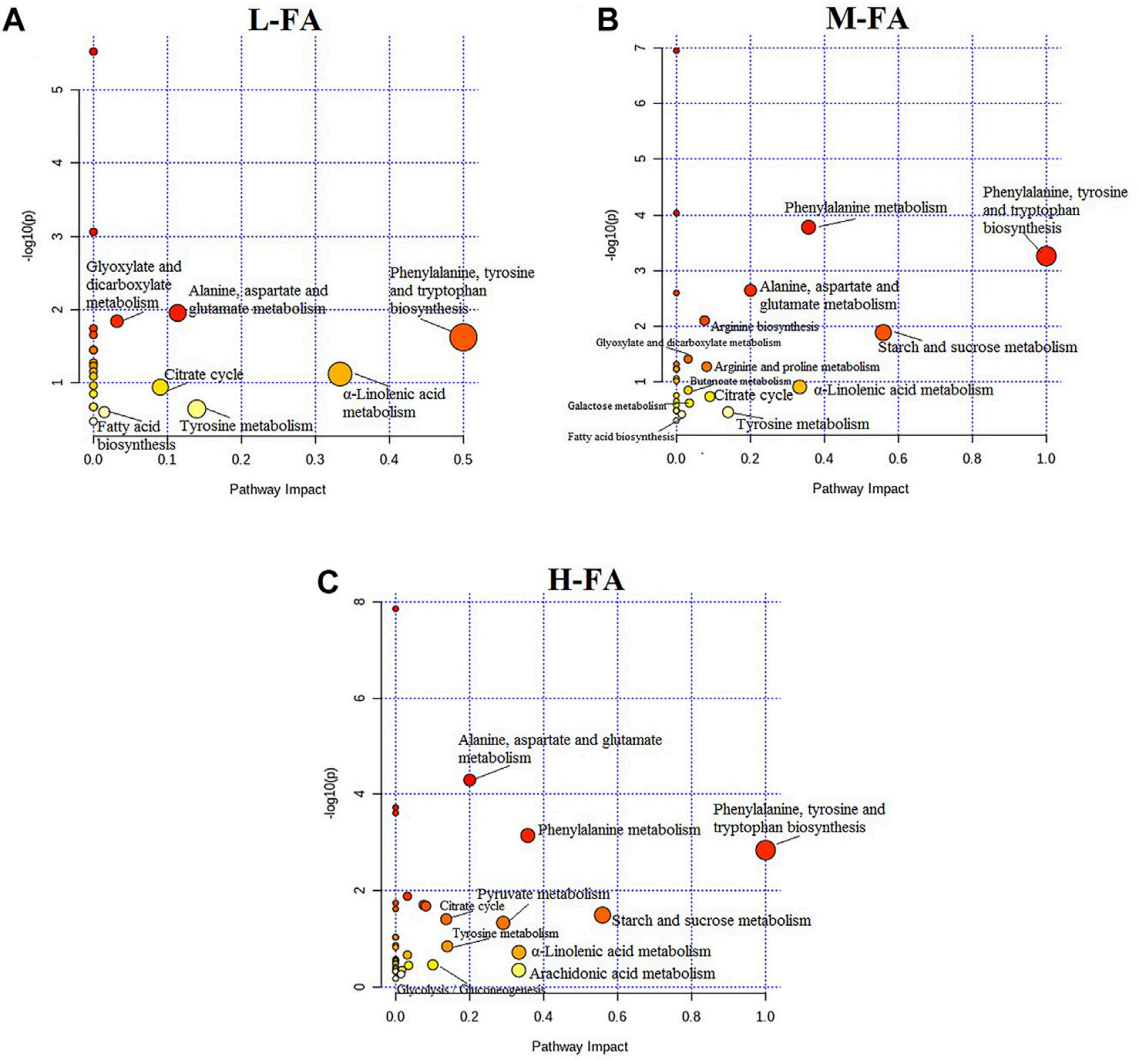
The pathways associated with differential metabolites in response to the low, middle, and high dosage treatment of folic acid.

Not only could metabolomic profiling disclose the up-/downregulation of individual metabolites, but it also offers a comprehensive view of metabolic pathway changes caused by MACO exposures and FA. From MetaboAnalyst software analysis of these discriminating metabolites, pathways with an impact value of greater than 0.1 were deemed as the main metabolic pathways. In the bubble plot in Figures 5A–C, low dosage of FA treatment was mainly related to seven pathways, namely, phenylalanine, tyrosine, and tryptophan biosynthesis; α-linolenic acid metabolism; tyrosine metabolism; alanine, aspartate, and glutamate metabolism; citrate cycle; glyoxylate and dicarboxylate metabolism; and fatty acid biosynthesis. Middle dosage of FA treatment further adjusts starch and sucrose metabolism, phenylalanine metabolism, arginine and proline metabolism, arginine biosynthesis, galactose metabolism, and butanoate metabolism on the basis of low dosage of FA effect.

High dosage of FA treatment achieves the maximization for IS rats associated with significantly altered pathways, including phenylalanine, tyrosine, and tryptophan biosynthesis; starch and sucrose metabolism; phenylalanine metabolism; α-linolenic acid metabolism; arachidonic acid metabolism; pyruvate metabolism; alanine, aspartate, and glutamate metabolism; tyrosine metabolism; citrate cycle; glycolysis/gluconeogenesis; arginine and proline metabolism; arginine biosynthesis; galactose metabolism; glyoxylate and dicarboxylate metabolism; butanoate metabolism; glycerophospholipid metabolism; and fatty acid biosynthesis. As shown in Supplementary Figure S1A, KEGG global metabolic network map metabolites in rats with IS under FA treatment are mostly related to amino acid pathway, organic acid pathway, fatty acids pathway, and carbohydrate metabolism, in keeping with results from common metabolomics and metagenomics studies. The relationships between genes and main metabolites of 48 brain tissue samples after FA treatment are shown in Supplementary Figure S1B, mainly involving regulating 21 metabolites and 1,199 genes, with arachidonic acid, palmitic acid, γ-aminobutyric acid, and arginine having a degree value of more than two hundred, and GCG, SLC16A10, SLC7A8, ALB, DECR1, and SLC6A19 genes having a degree value of more than six.

## Discussion

Compared with rats in the MOD group, the level of MDA, NO, TNF-α, IL-1β, IL-6, VEGF, HIF-1α, NOS, and MMP-2 in brain tissue decreased, and the level of SOD increased after FA administration. Oxygen free radical damage is one of the most important and certain factors in the occurrence and development of IS. The level of free radicals in the normal body remains relatively stable. When cerebral ischemia and hypoxia occur, the balance of production and clearance in the body is destroyed, resulting in the rapid production of free radicals and exhaustion of cholesterol content in cell membranes, which lead to the loss of integrity of nerve cell membranes, and eventually cell necrosis, cerebral edema, and other complications (Allen and Bayraktutan, 2009). As one of the main antioxidant enzymes, SOD blocks the toxic reaction of free radicals, removes superoxide anion free radicals, and protect cells from damage *via* catalyzing the disproportionation of superoxide anion free radicals. MDA that exists in the lipid peroxide produced by the action of free radicals on unsaturated fatty acids in the cell membrane is the metabolic end product of lipid peroxidation, which indirectly reflects the damage of oxygen free radicals in the cell organism (Liu et al., 2012).

NO is a messenger in the cell with free radical chemical properties, a simple structure, unstable traits, and a short half-life. The vasodilator effect of NO is so short that it loses brain protection after more than 2 h. Within 2–6 h of cerebral ischemia, NO mainly comes from iNOS, which is harmful to the ischemic tissue and can aggravate the damage. In the late stage of cerebral ischemia, the ischemic tissue induces iNOS expression to produce a large amount of NO, which plays a major role in the delayed progressive neuronal damage, leading to tissue damage aggravation (Muresanu et al., 2019; Li C. et al., 2020). It is currently believed that NOS-mediated production is involved in the early brain damage of ischemia, which directly released NO with neurotoxicity and is also the main mediator of glutamate excitotoxicity during ischemia and hypoxia. In the early stage of inflammation, white blood cells can be activated to release toxic oxygen metabolites, among which TNF-α and IL-1β are closely related to cerebral ischemia injury. After cerebral ischemia, serum TNF-α and IL-1β content were increased, which can promote the increase of vascular permeability, induce the expression of various inflammatory mediators, damage the blood–brain barrier function, form cerebral edema, and aggravate stroke injury (Sánchez-Gloria et al., 2021; Chen et al., 2015). Some studies have shown that microglia stimulated to activate IL-1β can produce and release a series of neurotoxic substances such as TNF-α and iNOS, which can aggravate the inflammatory response and cause secondary brain damage. In addition, IL-6 secreted by microglia enhances excitotoxicity by promoting Ca^2+^ influx to inflammatory aggravation (Sun et al., 2017). VEGF is a vital regulator of angiogenesis and provides nutritional support for nerves. Studies have shown that the expression of VEGF in neurons, astrocytes, microglia, and blood vessels of MCAO rats is significantly upregulated. Vascular regulation originates from HIF-1α in endothelial cells, which can promote the formation of new blood vessels in order to protect vascular endothelial cells, nourish neurons, and improve stroke symptoms and prognosis (Wang et al., 2020). In the early stage of IS, the content of VEGF and HIF-1α in the tissues was increased due to the self-protective response of the body; meanwhile, IL-6 and IL-1β release was increased due to inflammatory damage. Matrix metalloproteinases (MMPs) are one of the proteases that degrade the extracellular matrix, and their coding genes have homology. As a classic member of MMPs, MMP-2 can be secreted by a variety of cells in the body. After activation, it can effectively degrade type IV collagen and fibronectin, which has an important impact on cell or tissue repair. It also regulates blood coagulation factors and complement. When the expression increases, it can give rise to the disorder of the fibrinolysis system and increase the incidence of cerebrovascular disease (Fatar et al., 2005; Scrimgeour et al., 2020).

Cerebral ischemia and hypoxia cause physical damage to brain tissues and affect its biochemical and metabolic functions, which was manifested by abnormal fluctuations in the content of metabolites such as amino acids, fatty acid, and lipids. From the perspective of metabonomics, the changes constitute the metabolic characteristics of IS. In clinical practice, high levels of blood lipids, glucose, and cholesterol are common features of patients with IS. When stroke occurs, brain tissue edema, inflammation, neuronal damage, and infarction lead to significant alterations in the metabolites, especially free amino acid metabolism in brain tissue (Zhang A. H. et al., 2018). Excitatory amino acids with strong cytotoxicity that play an important role in pathophysiology of many diseases can damage cell membranes, mitochondria and lysosome membranes, and intracellular micro-organisms, and improve cytokine toxicity (Brouns et al., 2010). Due to the blood–brain barrier, glutamate as an important substance in the cerebrospinal fluid cannot be directly supplied to the brain through the blood. It must be synthesized in the brain through biochemical pathways to complete brain metabolism. The content of glutamate in brain tissue increases when IS occurs. The glutaminase inhibitor 6-diazo-5-oxo-L-norleucine can reduce the release of glutamate, and the abnormal content of glutamine in patients is likely to activate the self-protection mechanism of the central nervous system (Khanna et al., 2015; da Silva-Candal et al., 2018).

Lysine, which can pass through the blood–brain barrier, is one of the essential amino acids of the human body that help the blood–brain barrier in maintaining good permeability and promoting the growth of brain tissue. With the destruction of the blood–brain barrier, the content of lysine in cerebrospinal fluid is obviously abnormal (Goulart et al., 2019). As one of the body’s essential amino acids, phenylalanine produces tyrosine under the catalysis of phenylalanine lightase enzyme. After cerebral ischemia and reperfusion, the biosynthesis of catecholamines increases due to their large expression, which accumulates in large amounts in the brain and diffuses to the blood, cerebrospinal fluid, and neighboring neurons. Catecholamines damage the function of neurons, promote the development of neurons, and impair learning and memory through various mechanisms. The increasing excretion of phenylalanine in brain tissue from the MOD group stimulates an increase in the amount of tyrosine. Due to the catalyzing of tyrosine lightase and dopamine lightase, a large amount of catecholamines were produced in brain tissue, which can further aggravate cerebral ischemia (Gao et al., 2018; Qiu et al., 2020).

Hippuric acid is one of the metabolites of phenylalanine, and a significant reduction was observed in the MOD group, which may be due to the reduced consumption of phenylalanine. Previous studies have shown that the phenylalanine levels of subjects with stroke increased significantly compared with healthy controls, indicating that the phenylalanine metabolism pathway of stroke has been disturbed. Compared with the SHA group, the two branched chain amino acids (BCAAs) leucine and isoleucine in the MOD group are greatly reduced, which may be caused by the consumption of citric acid to reactivate brain function or used as cell signaling molecules as an important energy source of citrate cycle (Wang et al., 2017). Valine promotes normal growth of the body, repairs tissues, regulates blood sugar, and provides needed energy together with isoleucine and leucine (Liepert et al., 2015). Arginine is a biosynthetic substrate of nitric oxide (NO). The level of arginine was increased, suggesting that NO biosynthesis is affected by the feedback of the anaerobic state of the brain. Moreover, arginine and its related metabolites, such as asymmetric dimethylarginine and guanidinoacetate, have been shown to be potential biomarkers of cardiovascular disease related to aortic atherosclerosis (Csecsei et al., 2019). Homocysteine is an important biomarker for the pathophysiology of IS, which is also a sulfhydryl product of methionine metabolism. An increase in methionine can lead to high levels of cysteine, which can cause stroke (Gungor et al., 2018). Serine and alanine are fuel substrates in energy metabolism of brain cells to meet their high energy requirements, which can be converted into pyruvate by glycolysis to provide energy. As the product of glutamate metabolism in the glutamate/glutamine neurotransmitter cycle, GABA is the main inhibitory neurotransmitter. It is reported that cerebral ischemia can stimulate the synthesis of GABA and inhibit transamination leading to the level increasing, which was also confirmed in this study (Hosinian et al., 2016).

The brain as the main consumer of carbohydrates and oxygen has no long-term energy storage. MCAO-induced IS blocks the supply of oxygen and glucose in the brain and disrupts the tricarboxylic acid (TCA) cycle, in which the energy supply mode of the brain changes from aerobic metabolism to anaerobic metabolism. The increased anaerobic glycolysis of glucose leads to the increasing production of lactic acid to bring out slight acidosis, deemed as a limited ischemic protection (Lazzarino et al., 2019). However, the ATP produced by glycolysis with low energy efficiency cannot meet the energy needs of the brain. ATP needs to be catabolized into adenosine, which is then broken down into inosine, hypoxanthine, and xanthine to provide more energy. Creatine and creatine phosphate play an important role in maintaining a constant ATP level through the creatine kinase reaction (Geng et al., 2019; Nukui et al., 2020). Ketone bodies such as α-hydroxybutyrate can also be used as fuel for supplying the insufficient energy in the brain, which are transferred from the serum to the brain. The decreased content of 6-phosphate glucose and ribose indicates that insufficient glucose and oxygen supply leads to a decreased state in glycolysis and the pentose phosphate pathway (PPP) when the blood flow is restricted. The level of citrate is also reduced because almost no pyruvate can enter the TCA cycle. The reperfusion after ischemia bringing oxidative stress to neurons is the most significant feature (Belisário et al., 2016; Narne et al., 2017). Arachidonic acid and docosahexaenoic acid levels are elevated in the MOD group, which may be due to the peroxidation of cell membranes caused by the production of ROS to promote the release of cell membrane phospholipids (Ueno et al., 2018). Choline/phosphocholine is the precursor of all membrane phospholipases, which are used as a self-repair mechanism to build damaged membranes, leading to their serum levels being reduced in stroke rats (Sjöberg et al., 2009).

Fatty acids such as palmitoleic acid and palmitic acid showed elevated levels in the MOD group. Some studies have reported that α-linolenic acid can exert an effective neuroprotective effect on focal and global ischemia in animal models by glutamate-mediated excitatory neurons (Bork et al., 2018). The downregulation level of LysoPC such as LysoPC (17:0), LysoPC (20:1), and LysoPC (20:3) may lead to changes in the composition of glycerophospholipids in nerve membranes, and then impair phospholipid metabolism in meninges from the MOD group (Satoh et al., 1992; Liu et al., 2018). Acetylcarnitine as an intermediate in the β-oxidation of fatty acids has the ability to transport fatty acids to the cartilage membrane; the increased level in brain tissue of patients with IS suggests an increase in energy demand due to negative feedback from cerebral hypoxia (Seo et al., 2018). Taurine, inositol, and sphingosine are the main osmotic pressure control agents in the brain. The decrease in the content of creatine from brain indicates that IS disturbs ion balance and eventually generates brain edema. In addition to the osmotic regulation function, taurine can also act as a neuroprotective agent through several different mechanisms that inhibit cell apoptosis and oxidation (Menzie et al., 2013; Liang et al., 2016b; Li Y. J. et al., 2020). During the IS process, FA can reverse the reduced level of these metabolites related to osmotic regulation.

## Conclusion

In this study, we used a UPLC-MS-based metabolomics approach to explore the protective effect and mechanism of FA on MACO-induced IS rats. It found that FA could inhibit oxidative stress, reduce inflammation, promote angiogenesis and vasodilation, and accelerate cell reconstruction by regulating the altered biomarkers that are involved in amino acid metabolism, carbohydrate metabolism, fatty acid metabolism, citrate cycle (TCA cycle), organic acid metabolism, pyruvate metabolism, etc. This work also showed that the integrated metabolomic screening platform could be used to discover the potential biomarkers and reveal the protective effect and mechanism of natural products.

## Data Availability

The original contributions presented in the study are included in the article/Supplementary Material, further inquiries can be directed to the corresponding author.
