# Supplementary material for: An Integrated Metabolomic Screening Platform Discovers the Potential Biomarkers of Ischemic Stroke and Reveals the Protective Effect and Mechanism of Folic Acid
Source: Front Mol Biosci. 2022 May 18;9:783793. doi: 10.3389/fmolb.2022.783793 (PMC9158342; doi:10.3389/fmolb.2022.783793)
Supplement: Supplementary file 1 [file DataSheet1.docx]

**Table S1** Potential biomarkers changes in MCAO-induced ischemic stroke rats before and after folic acid treatment

| **No.** | **Classification** | **Chemical name** | **HMDB ID** | **Chemical formula** | **Ion mode** | **m/z** | **RT(min)** | **Trend changes of groups** | | | |
| --- | --- | --- | --- | --- | --- | --- | --- | --- | --- | --- | --- |
|  |  |  |  |  |  |  |  | **MOD** | **L-FA** | **M-FA** | **H-FA** |
| 1 | Amino acid | γ-Aminobutyric acid | HMDB00112 | C4H9NO2 | M+H | 104.0709 | 0.21 | **↑** |  | **√** | **√** |
| 2 | Amino acid | Lysine | HMDB00182 | C6H14N2O2 | M-H | 145.0974 | 0.55 | **↓** | **√** | **√** | **√** |
| 3 | Amino acid | Glycine | HMDB00123 | C2H5NO2 | M-H | 74.0249 | 0.91 | **↑** |  |  |  |
| 4 | Amino acid | Glutamate | HMDB60475 | C5H9NO4 | M-H | 146.0464 | 1.05 | **↑** | **√** | **√** | **√** |
| 5 | Organic acid | Taurine | HMDB00251 | C2H7NO3S | M+H | 126.0234 | 1.23 | **↓** |  |  |  |
| 6 | Carbohydrate | Ribose | HMDB00283 | C5H10O5 | M-H | 149.0446 | 1.44 | **↓** |  |  | **√** |
| 7 | α-keto acid | Pyruvic acid | HMDB00243 | C3H4O3 | M-H | 87.0079 | 1.85 | **↓** |  |  | **√** |
| 8 | Amino acid | Valine | HMDB00883 | C5H11NO2 | M-H | 116.0705 | 2.12 | **↓** | **√** | **√** | **√** |
| 9 | Organic acid | Creatine | HMDB00064 | C4H9N3O2 | M+H | 132.0777 | 2.22 | **↓** |  |  |  |
| 10 | Amino acid | Methionine | HMDB00696 | C5H11NO2S | M-H | 148.0427 | 2.72 | **↑** |  |  |  |
| 11 | Amino acid | Leucine | HMDB00687 | C6H13NO2 | M-H | 130.0862 | 2.98 | **↓** | **√** | **√** | **√** |
| 12 | Amino acid | Tyrosine | HMDB00158 | C9H11NO3 | M+H | 182.0813 | 3.11 | **↑** | **√** | **√** | **√** |
| 13 | Carbohydrate | Glucose | HMDB00122 | C6H12O6 | M+H | 181.0715 | 3.43 | **↓** |  | **√** | **√** |
| 14 | Amino acid | Isoleucine | HMDB00172 | C6H13NO2 | M+H | 132.1018 | 3.62 | **↓** |  |  | **√** |
| 15 | Organic acid | Phosphocreatine | HMDB01511 | C4H10N3O5P | M+H | 212.1131 | 3.78 | **↓** |  |  |  |
| 16 | Organic acid | Citric acid | HMDB00094 | C6H8O7 | M-H | 191.1235 | 4.25 | **↓** | **√** | **√** | **√** |
| 17 | Carbohydrate | Glucose 6-phosphate | HMDB01401 | C6H13O9P | M+H | 261.0355 | 4.37 | **↓** |  | **√** | **√** |
| 18 | Amino acid | Serine | HMDB00187 | C3H7NO3 | M+H | 106.0954 | 4.58 | **↑** |  |  |  |
| 19 | Organic acid | Lactic acid | HMDB01311 | C3H6O3 | M+H | 113.0219 | 4.71 | **↑** |  |  | **√** |
| 20 | Aminoacetic acid | Hippuric acid | HMDB00714 | C9H9NO3 | M+H | 180.0645 | 5.03 | **↓** |  | **√** | **√** |
| 21 | Amino acid | Phenylalanine | HMDB00159 | C9H11NO2 | M+H | 166.0846 | 5.41 | **↑** |  | **√** | **√** |
| 22 | Amino acid | Glutamine | HMDB00641 | C5H10N2O3 | M-H | 145.0609 | 5.63 | **↑** | **√** | **√** | **√** |
| 23 | Organic acid | α-Hydroxybutyrate | HMDB00008 | C4H8O3 | M+H | 105.1050 | 5.76 | **↑** |  |  | **√** |
| 24 | Fatty acid | Arachidonic acid | HMDB01043 | C20H32O2 | M-H | 303.2316 | 6.02 | **↑** |  |  | **√** |
| 25 | Organic base | Choline | HMDB00097 | C5H14NO | M-H | 103.1708 | 6.11 | **↓** |  |  |  |
| 26 | Amino acid | Homocysteine | HMDB00742 | C4H9NO2S | M+H | 136.1849 | 6.29 | **↑** |  |  | **√** |
| 27 | Amino acid | Arginine | HMDB00517 | C6H14N4O2 | M+H | 175.1210 | 6.40 | **↑** |  | **√** | **√** |
| 28 | Lipids | LysoPC(20:3) | HMDB10394 | C28H52NO7P | M+H | 546.3567 | 6.66 | **↓** |  |  | **√** |
| 29 | Amino acid | Alanine | HMDB00161 | C3H7NO2 | M+H | 90.0923 | 7.00 | **↑** |  |  | **√** |
| 30 | Lipids | LysoPC(17:0) | HMDB12108 | C25H52NO7P | M-H | 508.3422 | 7.14 | **↓** |  |  | **√** |
| 31 | Fatty acid | Palmitoleic acid | HMDB03229 | C16H30O2 | M+H | 277.2129 | 7.27 | **↓** |  |  |  |
| 32 | Fatty acid | Palmitic acid | HMDB00220 | C16H32O2 | M+H | 279.2316 | 7.57 | **↓** | **√** | **√** | **√** |
| 33 | Sphingolipid | Sphingosine | HMDB00252 | C18H37NO2 | M+H | 300.2901 | 7.73 | **↓** |  |  |  |
| 34 | Lipids | LysoPC(20:1) | HMDB10391 | C28H56NO7P | M+H | 550.3854 | 8.12 | **↓** |  |  | **√** |
| 35 | Fatty acid | α-linolenic acid | HMDB01388 | C18H30O2 | M+H | 279.2317 | 8.23 | **↑** | **√** | **√** | **√** |
| 36 | Fatty acid | Docosahexaenoic acid | HMDB02183 | C22H32O2 | M+H | 329.2458 | 8.54 | **↑** |  |  |  |
| 37 | Amino acid | Acetylcarnitine | HMDB00201 | C9H18NO4 | M-H | 203.2435 | 8.66 | **↑** |  |  |  |


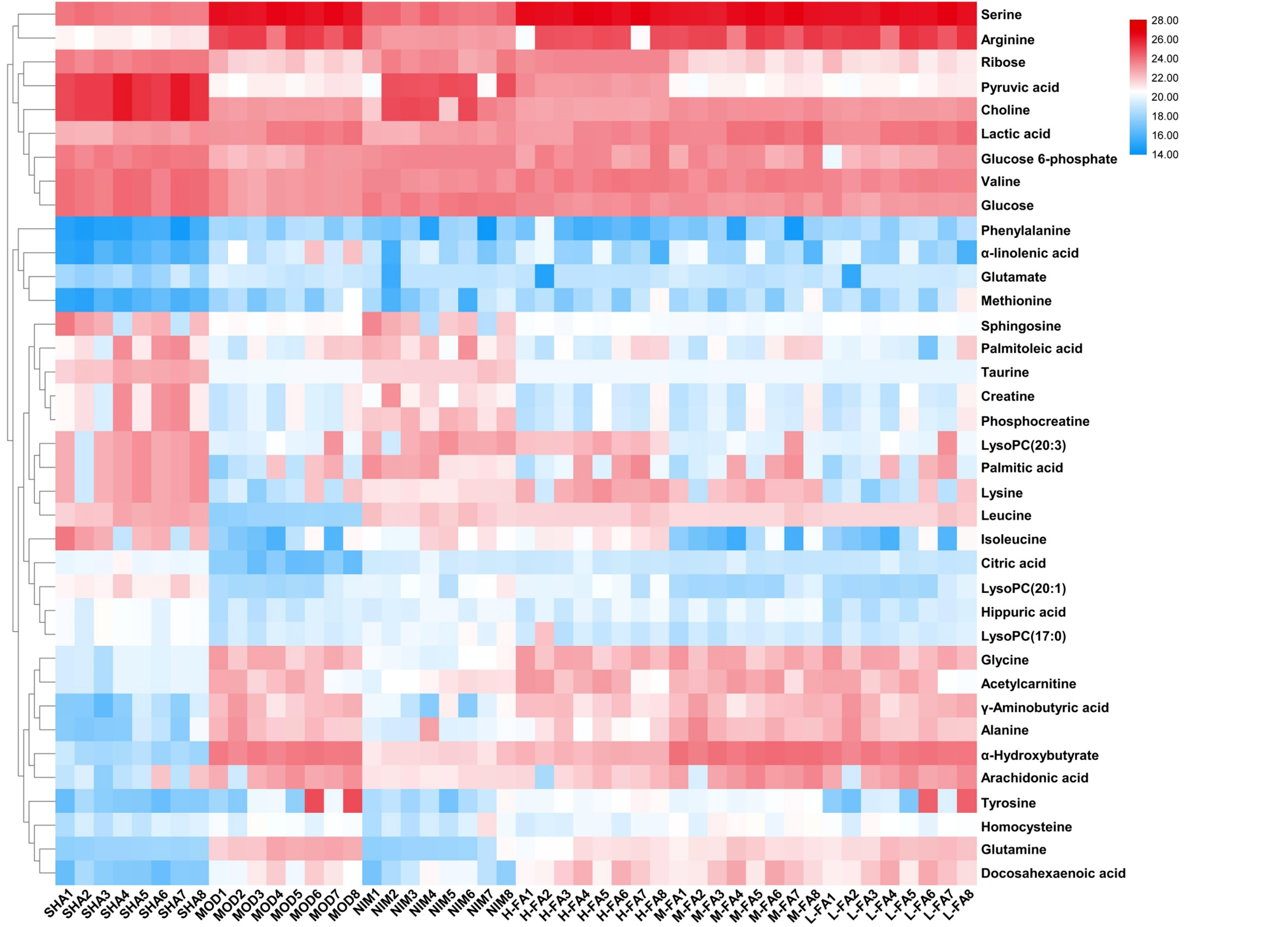


**Figure S1** Heatmap illustrating the metabolites changes in brain tissue of SHA, MOD, NIM, L-FA, M-FA and H-FA group from minimum ( blue) to maximum ( red) values.


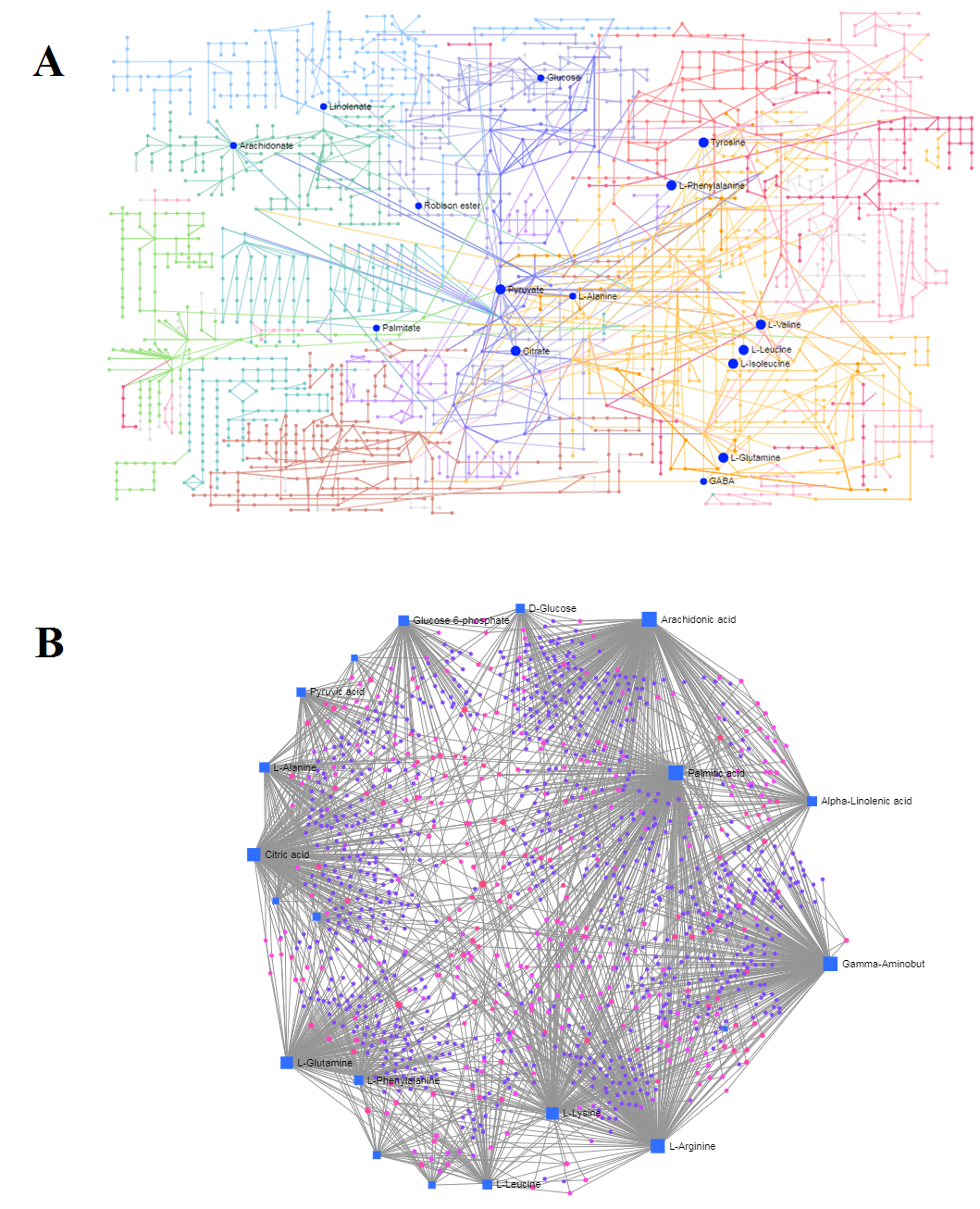


**Figure S2** The detailed KEGG pathway involved in metabolites associated with folic acid treatment (A). The relationships of genes and main metabolites of brain tissue sample after folic acid treatment (B).
